# Renal transcriptome profiles in mice reveal the need for sufficient water intake irrespective of the drinking water type

**DOI:** 10.1038/s41598-022-14815-5

**Published:** 2022-06-28

**Authors:** Woo-Jeong Shon, Mi-Na Park, Jooyoung Lee, Ji-Hee Shin, Dong-Mi Shin

**Affiliations:** 1 Department of Food and Nutrition, Seoul National University College of Human Ecology, Gwanak-gu, Seoul, 08826 Republic of Korea; 2 Research Institute of Human Ecology, Seoul National University, Gwanak-gu, Seoul, 08826 Republic of Korea

**Keywords:** Molecular biology, Molecular medicine

## Abstract

This study sought to characterize the impact of long-term dehydration in terms of physiological and biochemical parameters, as well as renal transcriptomes. Furthermore, we assessed whether consumption of specific types of water elicit more beneficial effects on these health parameters. To this end, C57BL/6 mice were either provided water for 15 min/day over 2 and 4 weeks (water restricted; RES), or ad libitum access to distilled (CON), tap, spring, or purified water. Results show that water restriction decreases urine output and hematocrit levels while increasing brain vasopressin mRNA levels in RES mice compared to control mice (CON). Meanwhile, blood urea nitrogen and creatinine levels were higher in the RES group compared to the CON group. Kidney transcriptome analysis further identified kidney damage as the most significant biological process modulated by dehydration. Mechanistically, prolonged dehydration induces kidney damage by suppressing the NRF2-signaling pathway, which targets the cytoprotective defense system. However, type of drinking water does not appear to impact physiological or blood biochemical parameters, nor the renal transcriptome profile, suggesting that sufficient water consumption is critical, irrespective of the water type. Importantly, these findings also inform practical action for environmental sustainability by providing a theoretical basis for reducing bottled water consumption.

## Introduction

The human body relies heavily on water to perform most biological and physiological functions, including nutrient transport, body temperature regulation, breaking down organic and inorganic materials, lubricating joints and internal organs, and providing structure to cells and tissues^1,2^. Consequently, for optimal body function, sufficient hydration is required^3^. On average, it is recommended that a sedentary adult drink at least 1.5 L of water per day^1^; however, available data on typical water consumption show a high prevalence of inadequate hydration^4^. In fact, more than 75% of adults do not achieve water intake recommendations^4,5^.

Dehydration is an adverse consequence of inadequate water intake, fluid loss, or both^6^. The impact of dehydration on health has been researched with regard to physical and cognitive functions^7,8^, with a particular focus on the role of dehydration in the physical actiity of athletes and military personnel^9^. Under mild levels of dehydration, individuals engaging in rigorous physical activity experience decreases in performance related to reduced endurance, increased fatigue, and altered thermoregulatory capability^10^. Additionally, mild dehydration can disrupt mood and cognitive function^11^. Mild to moderate levels of dehydration can impair performance in various functions, such as short-term memory, perceptual discrimination, arithmetic ability, motor tracking, and psychomotor skills^8,12^. Most studies on dehydration have focused on either very young (infancy) or old (elderly) individuals or athletes at a time when sufficient water intake is absolutely critical. However, adults who consume less water than recommended, and who tend to drink beverages containing natural diuretics, such as coffee, green tea, and alcohol may also experience excess fluid loss^6^ ,thus increasing the likelihood of chronic dehydration^2^. Chronic dehydration results from less than adequate rehydration of daily water losses over a period of time^2^. In a study on the hydration status of university students, 46% of the students were dehydrated, while 59% had inadequate water intake and were more likely to become dehydrated via coffee intake^13^. Despite the high risk of chronic dehydration in adults, previous studies have elucidated the effects of dehydration based on acute deprivation dehydration of one or two days, while little is known regarding the negative health effects of prolonged dehydration during this period of life.

The kidneys are responsible for regulating whole-body water homeostasis, guided by the antidiuretic hormone vasopressin, which is secreted by the posterior pituitary in response to changes in plasma osmolality^14^. Classical dehydration results in a pre-renal state associated with intrarenal vasoconstriction but with the relative maintenance of the glomerular filtration rate (GFR)^15^. When water depletion is severe, the GFR drops, however can be fully recovered via hydration^16^. Therefore, dehydration is not considered a risk factor for kidney injury. Meanwhile, recent epidemiological evidence suggests that chronic dehydration might be a risk factor for the development and progression of chronic kidney disease (CKD)^17^, as highlighted in studies on a mysterious form of CKD in Central America^18^. Dehydration was frequently observed in most patients with the disease, despite the absence of major CKD etioloigc factors, such as high blood pressure and diabetes^19^. Although the complete etiology of the disease remains unknown, a powerful hypothesis is that it may be correlated with chronic dehydration. However, the underlying mechanisms through which chronic dehydration negatively affects the physiological function of the kidneys have not been fully identified.

Drinking water quality as well as quantity are crucial factors for health because of environmental pollution, different mineral contents, and the presence of toxic substances in water^20^. Municipal water refers to tap water (TAP) that is sent to various industries and homes through underground pipes^21^. However, the dearth of safe and accessible drinking water in many developing countries, resulting in increased concerns regarding the health effects related to harmful components in municipal drinking water, has prompted the consumption of commercial bottled water (BOT)^20^. While there are many different kinds of commercial BOT, spring water (SPR) is the most common kind of BOT^22,23^. It comes from groundwater, which is water that exists underground in an aquifer that sits at or below the earth’s natural water table. In assessing drinking water quality, consumers perceive BOT as pure, safe, convenient, and as having a good taste; thus, there has been a significant increase in its consumption, despite its excessively high prices compared with TAP^24^. Accordingly, the BOT industry is growing rapidly, with consumption reaching a record high of USD 217.66 billion in 2020; the industry is expected to expand at a compound annual growth rate of 11.1% from 2021 to 2028^25^. However, concern over the environmental impact of the production and disposal of plastic bottles and consequent greenhouse gas emissions is emerging^26^. In addition, multiple studies have found that the BOT is frequently contaiminated with high levels of microplastics (MP)—small pieces of plastic debris < five millimeters^27–29^. In fact, the rported MP levels in BOT range from 1.4 MP/L to 5.42E + 07 MP/L^30,31^, with higher concentrations generally detected in BOT compared to TAP^30^. Exposure to MPs may lead to potential associated risks to human health^30^. Hence, given the risk to environmental sustainability and human health, it is necessary to persuade the public to adopt and maintain more sustainable behaviors, such as drinking TAP instead of BOT. Implementation of water purification systems (PUR) that filter out bacteria, chlorine, and other unpleasant taste elements, may further encouarge the switch from BOT^32^. To date, most studies on drinking water have been focused on comparing the safety or mineral contents of drinking water. However, the differential health-related physiological responses resulting from the consumption of drinking water with different types have not yet been studied.

Accordingly, the main objective of the present study is to investigate the effects of prolonged dehydration, due to inadequate water intake, on changes in physiological and biochemical parameters, as well as the kidney transcriptome. Additionally, differential physiological and transcriptional changes resulting from the consumption of four different types of drinking water (distilled water [DW] generated through the distillation process, commercial bottled spring water [SPR], tap water from city (TAP), and purified water [PUR]) was assessed. To achieve these aims, we (i) used a chronic dehydration-induced protocol in which mice had access to water for 15 min per day over a 2- and 4-week period, and (ii) tested four different types of drinking water in the murine model. The findings of this study may provide molecular-level evidence for the importance of adequate hydration and could serve as a basis for decreasing the consumption of bottled water, which could substantially reduce the carbon footprint of humans.

## Results

### Changes in body weight, dietary intake, and water consumption after water restriction

**Figure 1:**
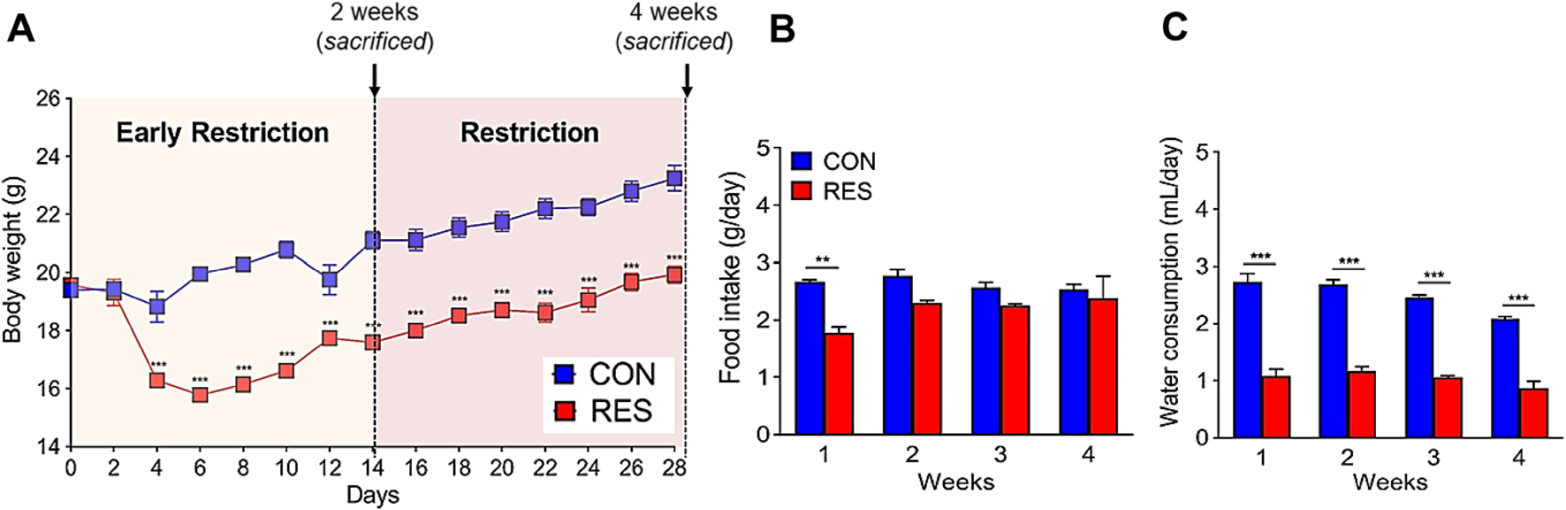
Effects of prolonged dehydration in mice. Changes in (**A**) body weight, (**B**) food intake, and (**C**) water consumption in hydrated (CON) and restricted (RES) mice (n = 10 mice/group). Data are expressed as mean ± SEM. Statistical significance was determined using a two-tailed Mann–Whitney test. *p < 0.05, **p < 0.01, ***p < 0.001.

Mice were provided limited access to a water bottle for 15 min per day for 2 weeks (early restriction, 2 W RES) and 4 weeks (4 W RES). The body weights of mice in the RES group were markedly decreased from day 0 to 6, showing a 25.0% reduction compared with that in CON mice with ad libitum water intake (Fig. 1A). After day 6, the body weight of RES mice was gradually restored, reaching 86.7% of that of CON mice at day 28; however, the difference in body weight between the RES and CON groups remained significant (*p* < 0.001). RES mice consumed 33% less feed within the first week, although consumption gradually increased, leading to the observation of no significant difference between the two groups (Fig. 1B). In contrast, water intake in the RES group was consistently and significantly lower than that in the CON group throughout the experimental period (*p* < 0.001; Fig. 1C), reaching approximately 50% that of the CON group on day 28. Thus, the dramatic weight reduction observed during week 1 may have resulted from a decrease in both diet and water intake, whereas in the final week, the decrease in water intake served as the main contributor to the observed body weight reduction.

### Assessment of physiological markers for dehydration status

**Figure 2:**
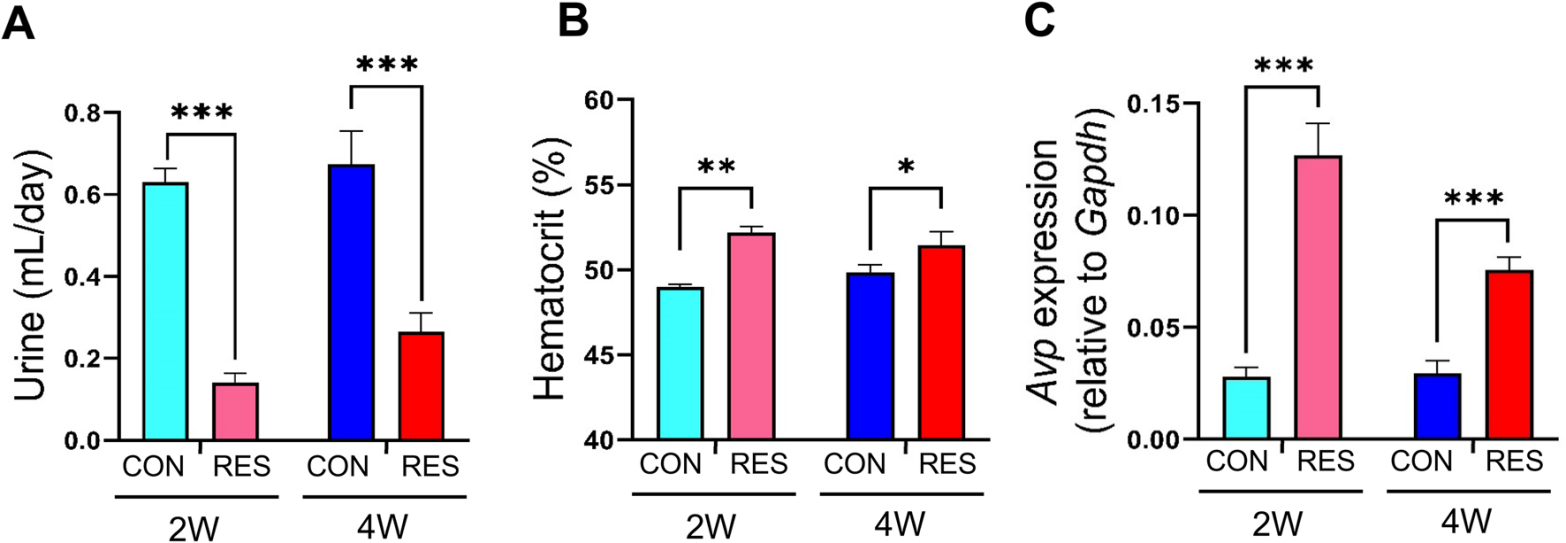
Changes in physiological markers corresponding to dehydration following prolonged water restriction. (**A**) The 24 h urine volume, (**B**) hematocrit level (%), and (**C**) mRNA expression of *Avp* (vasopressin) in the brain of hydrated (CON) and restricted (RES) mice (n = 10 mice/group). Data are expressed as means ± SEMs. Statistical significance was determined using a two-tailed Mann–Whitney test. *p < 0.05, **p < 0.01, ***p < 0.001.

To verify whether the reduction in water intake was sufficient to induce physiological changes in RES mice, we examined the level of physiological markers associated with dehydration status. Compared to the 2 W CON and 4 W CON mice, the mice in 2 W RES and 4 W RES showed a significant decrease in 24 h urine volume from 0.60 to 0.14 mL/day and 0.65 to 0.30 mL/day, respectively (*p* < 0.001; Fig. 2A); this result is indicative of normal physiological adaptation to reduced water intake. The hematocrit levels in the RES groups (2 W and 4 W RES) were substantially higher than those in the ad libitum controls (Fig. 2B). Furthermore, we measured the transcript levels of the antidiuretic hormone vasopressin, a posterior pituitary hormone encoded by the *Avp* gene, in the brain and found significant increases in vasopressin production in both 2 W and 4 W RES, compared to 2 W and 4 W CON, respectively (*p* < 0.001; Fig. 2C). These findings suggest that our water restriction protocol effectively induces physiological responses to dehydrated conditions.

### Changes in blood biochemical parameters after dehydration

**Figure 3:**
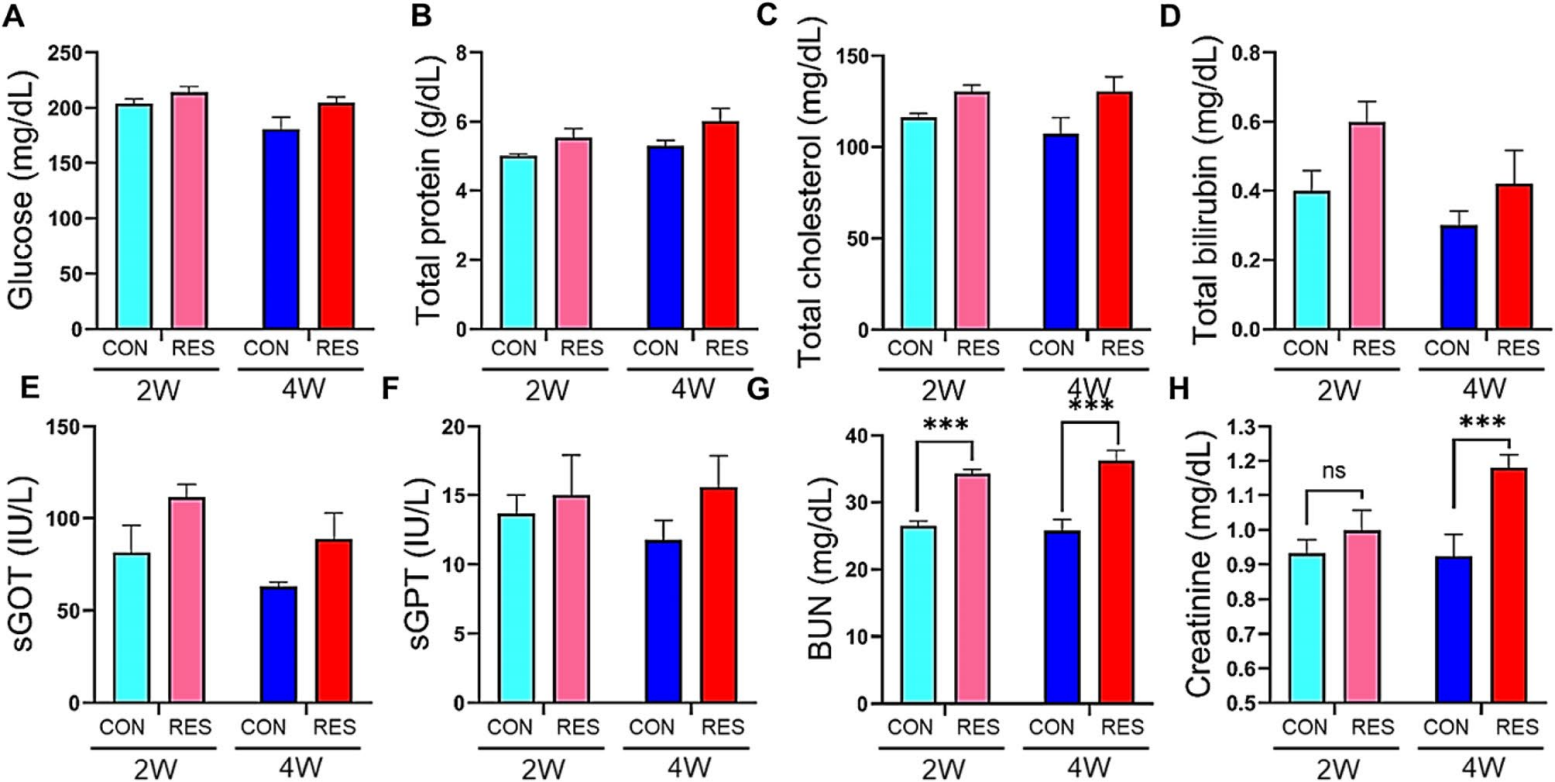
Changes in blood biochemical parameters following prolonged dehydration. (**A**) Glucose, (**B**) Total protein, and (**C**) Total cholesterol levels (n = 10 mice/group). Additionally, biochemical parameters for liver function, such as Total bilirubin (**D**), sGOT (**E**), and sGPT (**F**), and for kidney function, such as BUN (**G**) and Creatinine (**H**), in serum were measured (n = 10 mice/group). Data are expressed as means ± SEM. Statistical significance was calculated using a two-tailed Mann–Whitney test. *p < 0.05, **p < 0.01, ***p < 0.001.

To clarify the effect of dehydration on blood biochemical parameters, serum glucose, total protein, and total cholesterol levels were measured (Fig. 3A–C). Additionally, blood biochemical parameters, including total bilirubin, sGOT, and sGPT, were evaluated to determine liver function (Fig. 3D–F), while BUN and creatinine were evaluated to determine kidney function (Fig. 3G,H). Although all tested parameters did not exhibit a significant difference between the groups, they tended to be higher in the RES group (2 W and 4 W RES) compared to the CON group (2 W and 4 W CON), possibly owing to high blood osmolality in dehydrated mice. Notably, the BUN levels were significantly higher in both 2 W and 4 W RES, compared to 2 W and 4 W CON, respectively (*p* < 0.001; Fig. 3G). In addition, although not significant after 2-week dehydration (early restriction, *p* = 0.3879), mice inthe 4 W RES group had higher creatinine concentrations than 4 W CON mice (*p* < 0.001). BUN and creatinine are well-known markers of kidney damage^33^; a decline in kidney function due to kidney damage can cause an increase in BUN and creatinine levels. These findings prompted us to further assess the molecular mechanisms by which dehydration causes renal tissue damage.

### Global transcriptome profiling of kidney tissues in dehydrated mice

**Figure 4:**
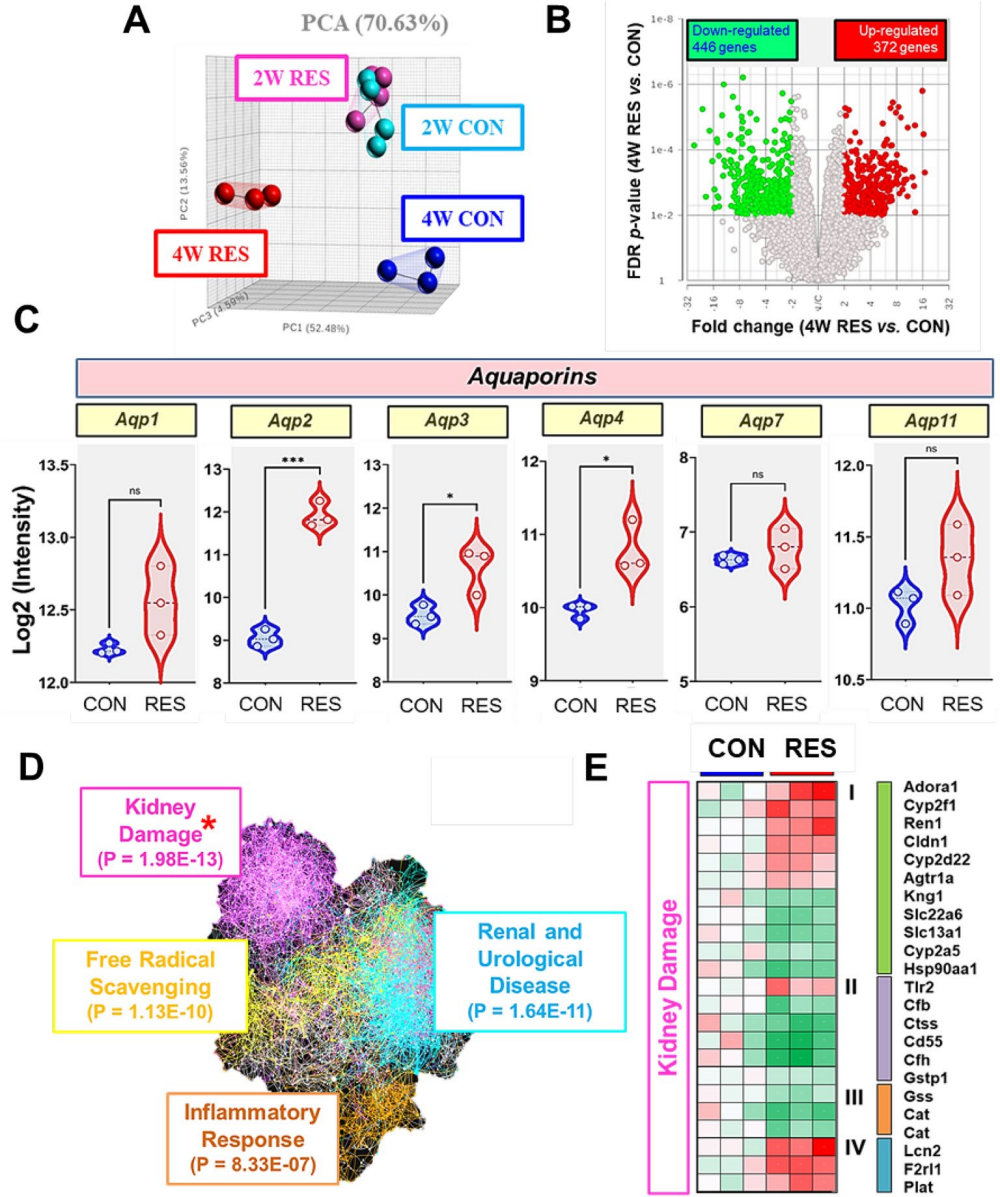
Global transcriptome profiling of kidney tissues in water-restricted and control mice. (**A**) A 3-D principal component analysis (PCA) plot of the renal transcriptome data represents profiles for each group: 2W EC (Turkish blue), 2W ER (Pink), 4W C (Blue), and 4W R (Red). (**B**) Volcano plot of all differentially expressed transcripts between the 4 W RES and C group. Significant genes were identified by performing *t*-tests (thresholds of FDR < 0.05) and twofold change restriction analysis. Upregulated and downregulated genes are represented in red and green, respectively. (**C**) Increased expression of *Aqp1, Aqp2, Aqp3, Aqp4, Aqp7*, and *Aqp11* in kidney tissue of dehydrated mice. (**D**) Functional classification of differentially expressed genes in dehydrated mice. Top disease and functional categories of gene sets among differentially expressed genes in dehydrated compared to control mice. GO terms with red asterisk indicate the most enriched biological functions in gene set enrichment analysis. (**E**) Heat map of kidney damage-related genes that are differentially expressed between the groups. The given values are the average of normalized intensities, with red representing higher levels of expression and green indicating lower levels. Data are expressed as mean ± SEM. Statistical significance was determined using a two-tailed Mann–Whitney test. *p < 0.05, **p < 0.01, ***p < 0.001.

To examine the molecular effects of prolonged dehydration on kidney damage, we performed gene expression profiling analysis using whole-genome arrays covering ~ 45,000 transcripts. Principal component analysis (PCA) showed that the gene expression profiles from 2 W RES and 2 W CON exhibited significnat crossover, whereas the transcriptome profiles from 4 W RES were readily distinguishable from those of 4 W CON (Fig. 4A). This finding implies that 4-week dehydration might have a greater effect on kidney function than early water restriction. Thus, further analysis focused on the 4-week dehydration group (4 W RES *vs.* 4 W CON). Differentially expressed genes in 4 W RES mice compared with 4 W CON mice were identified using *t*-tests (false discovery rate [FDR] < 0.05) and twofold change restriction analysis. A total of 818 probes were differentially identified, with 372 probes upregulated and 446 downregulated in the kidneys of RES mice (Fig. 4B). We first assessed the transcript levels of genes encoding aquaporin (*Aqp*) water channels, which are regulated by water balance in the body^34^. Of the eight aquaporin genes, *Aqp1*, *Aqp2*, *Aqp3*, *Aqp4*, *Aqp7*, and *Aqp11* were expressed at sufficient levels to allow the comparison of their expression in the kidneys of 4 W RES and CON mice (Fig. 4C). Notably, *Aqp2* expression, which is regulated by vasopressin or the physiological response to thirst^35^, was significantly increased in the 4 W RES group compared with the CON group (Fig. 4C, p = 0.007). Moreover, the expression levels of *Aqp3* and *Aqp4* were also significantly increased in the 4 W RES group (Fig. 4C, p = 0.036 and 0.023, respectively).

Differentially expressed genes in the RES group compared with the CON group were further classified based on their biological functions, and the significance of dehydration-responsive gene enrichment were examined for each disease and biological function category by Fisher’s exact test (Fig. 4D). The top four significant categories were kidney damage (*p* = 1.98 × 10^–13^), renal and urological disease (*p* = 1.64 × 10^–11^), free radical scavenging (*p* = 1.13 × 10^–10^), and inflammatory response (*p* = 8.33 × 10^–7^). Interestingly, all four categories were interconnected with the node of kidney damage in the classification analysis; therefore, we further examined kidney damage gene expression profiles (Fig. 4E). Among the 23 genes associated with kidney damage, those involved in normal physiological responses were labeled as Cluster I. As expected, the transcript levels of renin (encoded by *Ren*) and angiotensin II receptor (*Agtr1*) were significantly higher in dehydrated mice than in CON mice. Further, the expression of *Kng1*, which encodes a positive regulator of urine volume, was decreased in dehydrated mice, and genes that play important roles in innate or adaptive immunity, such as the complement system (i.e., *Cd55*, *Cfb*, and *Cfh*) and antigen presentation (i.e., *Ctss*), were markedly downregulated by dehydration (Cluster II). Notably, the transcript levels of antioxidant enzymes, including *Gss*, *Gstp1*, and *Cat*, which encode glutathione, glutathione S transferase, and catalase, respectively, were significantly downregulated in 4 W RES mice compared with CON mice (Cluster III). Additionally, kidney failure markers, namely *Lcn2* (lipocalin 2), *F2rl1* (coagulation factor 2 receptor-like 1), and *Plat* (plasminogen activator inhibitor-1), were upregulated (Cluster IV). These findings implied that long-term dehydration significantly altered renal transcriptional networks, ranging from normal physiological processes, such as water balance and detoxifying reactions, to pathophysiological processes, including inflammatory responses and tissue damage, emphasizing the physiological importance of sufficient water intake.

### Enriched canonical pathways in the kidneys affected by sustained dehydration

**Figure 5:**
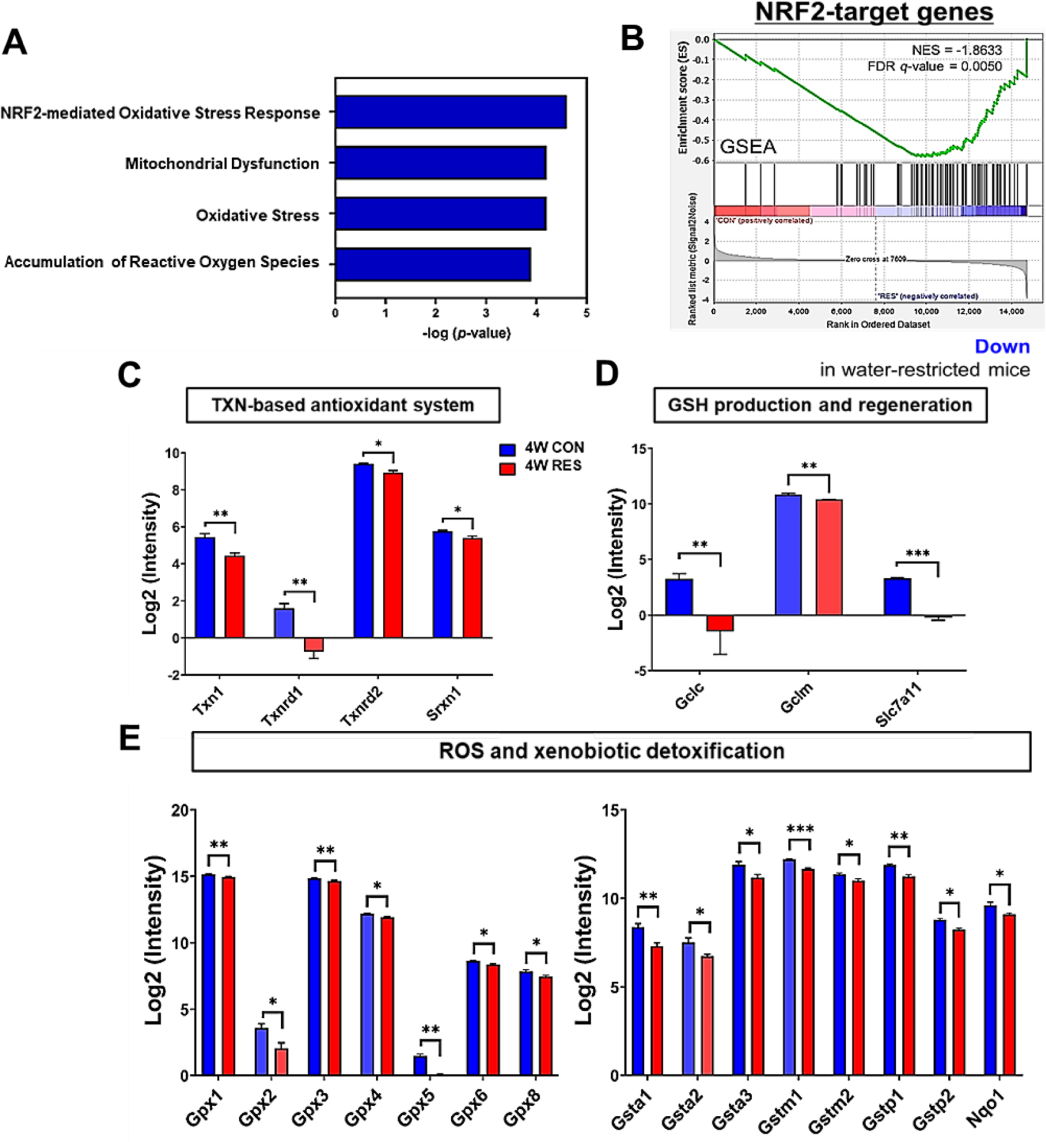
Prolonged dehydration exacerbates kidney damage by suppressing the NRF2-signaling pathway in mice. (**A**) Canonical pathway of gene sets among differentially expressed genes significantly detected in dehydrated compared to control mice. Statistical significance of pathway modulation was calculated via a right-tail Fisher’s exact test in Ingenuity Pathway and represented as –log(*p* value). (**B**) Gene set enrichment analysis (GSEA) was used to evaluate the most significant functional category of differentially expressed genes by dehydration in mice and generate enrichment plot of NRF2-target genes. (**C**–**E**) Bar graphs show the expression of downstream target genes of the NRF2-signaling pathway. (**C**) TXN-based antioxidant system, (**D**) GSH production and regeneration, and (**E**) ROS and xenobiotic detoxification are regulated by the NRF2-signaling pathway. Data are expressed as mean ± SEM. Statistical significance was calculated using a two-tailed Mann–Whitney test. *p < 0.05, **p < 0.01, ***p < 0.001.

To understand the implicit mechanism underlying kidney damage as a result of sustained dehydration, we confirmed the top significant pathways by canonical pathway analysis. We found that the NRF2-mediated oxidative stress response, mitochondrial dysfunction, oxidative stress, and accumulation of reactive oxygen species (ROS) were in the top four significant categories (Fig. 5A). Gene set enrichment analysis also revealed that the NRF2-downstream target genes constituted the most enriched biological process category regulated by prolonged dehydration and were significantly downregulated in the kidneys in the RES group (FDR *q* = 0.0050, Fig. 5B). NRF2 controls the expression of key components of the glutathione (GSH) and thioredoxin (TXN) antioxidant systems, as well as enzymes involved in ROS and xenobiotic detoxification, thus playing a fundamental role in maintaining the redox homeostasis of the cell^36^. Specifically, decreased expression of NRF2-downstream target genes, including TXN-based antioxidant system (*Txn1*, *Txnrd1*, *Txnrd2*, *Srxn1*) and GSH production- and regeneration-related molecules (*Gclc*, *Gclm*, *Slc7a11*), were detected in the 4 W RES group (Fig. 5C,D). In addition, a reduction in the transcriptional levels of numerous ROS-detoxifying enzymes, such as the glutathione peroxidase family (*Gpx1*, *Gpx2*, *Gpx3*, *Gpx4*, *Gpx5*, *Gpx6*, and *Gpx8*) and several glutathione S-transferases (Gsts; *Gsta1*, *Gsta2*, *Gsta3*, *Gstm1*, *Gstm2*, *Gstp1*, *Gstp2*, and *Nqo1*), were detected in the 4 W RES group (Fig. 5E). These results demonstrate that the NRF2-regulated protective defense system was dysregulated at the transcriptional level as a consequence of sustained dehydration.

### Effects of different drinking water types on biochemical markers and transcriptome profiles in kidney

Next, we explored whether, in addition to drinking water quantity, drinking water quality also affects the physiological responses of the body. Thus, we examined whether the consumption of different water types induce different physiological responses. We tested four types of drinking water in an in vivo system: DW (control), TAP, SPR, and PUR. Furthermore, to assess whether physiological response differ according to the type of drinking water, we evaluated these differences relative to the responses observed in dehydrated (DEH) mice. Mice were allowed to drink the assigned water for 4 weeks with free and full access to the water bottle, with the exception of mice in the DEH group. The contents of 23 minerals were determined for each experimental water sample (Supplementary Table S1). None of the tested minerals were detected in DW, whereas the other drinking water types had different levels of total mineral contents, with a rank order of SPR > TAP > > PUR. Food intake and water consumption were not affected by the water source (Table 1). Mice drank less TAP and more SPR; however, there were no significant differences in water intake between the DW group and other water source groups. Only the DEH group showed a significant decrease in water consumption compared with the experimental groups.

**Table 1:** Growth, dehydration status, and hematological index of the experimental groups by water source.

Variables	DW	TAP	PUR	SPR	DEH
**Body weight (g)**					
Initial	19.4 ± 0.25^NS^	19.2 ± 0.37	19.3 ± 0.28	19.2 ± 0.24	19.5 ± 0.18
Final	23.2 ± 0.44^a^	22.8 ± 0.62^a^	23.1 ± 0.48^a^	23.4 ± 0.39^a^	19.9 ± 0.30^b^
Food intake (g/day)	2.53 ± 0.09^NS^	2.53 ± 0.19	2.60 ± 0.17	2.75 ± 0.02	2.37 ± 0.39
Water consumption (g/day)	2.43 ± 0.15^a^	2.35 ± 0.13^a^	2.55 ± 0.11^a^	2.61 ± 0.09^a^	1.04 ± 0.06^b^
**Dehydration status**					
Urine (mL)	0.7 ± 0.08^a^	0.8 ± 0.03^a^	0.8 ± 0.18^a^	0.7 ± 0.09^a^	0.3 ± 0.03^b^
Hematocrit (%)	49.8 ± 0.47^NS^	50.3 ± 0.95	48.9 ± 0.79	49.3 ± 0.23	51.5 ± 0.71
*Avp* (Relative to *Gapdh*)	0.03 ± 0.01^a^	0.02 ± 0.00 ^a^	0.02 ± 0.00 ^a^	0.02 ± 0.01^a^	0.08 ± 0.01^b^
**Hematologic index**					
Glucose (mg/dL)	181.0 ± 10.78^NS^	209.3 ± 16.57	211.8 ± 13.57	217.0 ± 6.96	218.0 ± 5.21
Total protein (g/dL)	5.3 ± 0.20^NS^	5.7 ± 0.23	5.2 ± 0.09	5.1 ± 0.13	5.8 ± 0.40
Total cholesterol (mg/dL)	107.0 ± 9.07^NS^	119.0 ± 15.76	112.0 ± 9.28	107.0 ± 8.11	132.6 ± 9.70
Total bilirubin (mg/dL)	0.23 ± 0.03^NS^	0.40 ± 0.11	0.30 ± 0.04	0.20 ± 0.00	0.46 ± 0.14
sGOT (IU/L)	63.0 ± 2.48^NS^	80.8 ± 3.00	84.5 ± 11.70	115.8 ± 26.91	108.8 ± 15.11
sGPT (IU/L)	11.75 ± 1.44^NS^	13.00 ± 1.92	13.50 ± 2.06	18.50 ± 4.50	15.6 ± 2.25
BUN (mg/dL)	26.0 ± 1.65^a^	27.8 ± 2.21^a^	27.0 ± 1.47^a^	26.8 ± 0.48^a^	36.2 ± 1.50^b^
Creatinine (mg/dL)	1.00 ± 0.04^a^	1.05 ± 0.09^a^	1.15 ± 0.06^a^	1.05 ± 0.03^a^	1.30 ± 0.04^b^

*BUN* blood urea nitrogen, *DEH* dehydration, *DW* distilled water, *NS* not significant, *PUR* purified water, *sGOT* serum glutamic-oxaloacetic transaminase, *sGPT* serum glutamic pyruvic transaminase, *SPR* spring water, *TAP* tap water.

^a^Post-hoc statistically significant differences are represented by different letters.

Accordingly, urine volume and *Avp* transcript levels in the brain showed significant differences only in comparison with the DEH group, i.e., there were no significant differences among the water source experimental groups (Table 1). Moreover, most of the blood biochemical parameters did not differ significantly between groups. Concentrations of BUN and creatinine were only affected by DEH, although there were no significant differences among water source groups.

**Figure 6:**
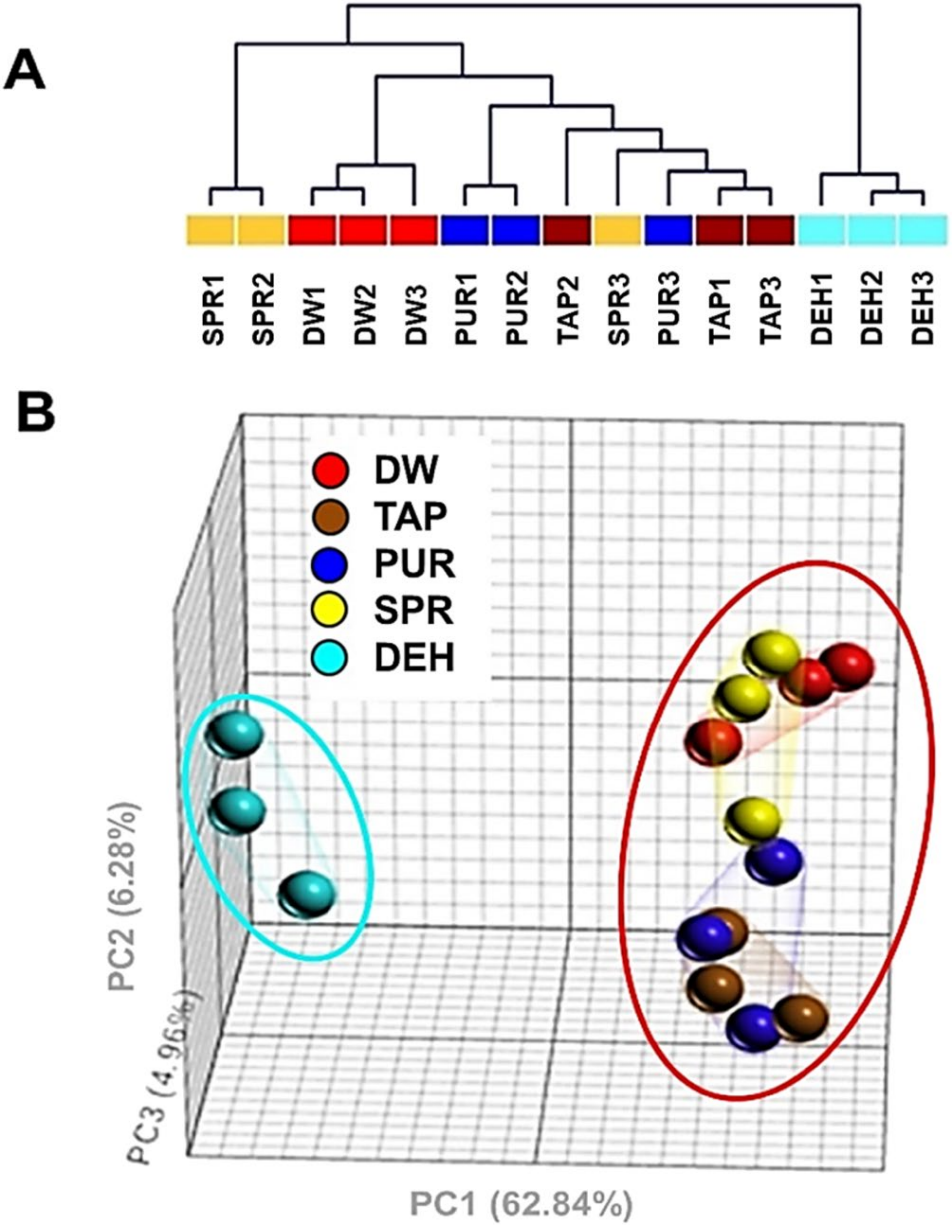
Effects of different drinking water types on transcriptome profiles in kidney. (**A**) Hierarchical clustering and (**B**) a 3-D view of principal component analysis (PCA) of differentially expressed genes (DEGs). Each color and ball represent the transcriptome corresponding to an individual mouse. The DEGs corresponding to the experimental groups were identified using one-way ANOVA (*p* < 0.05).

Next, gene expression profiling analysis using the mouse whole-genome array was performed to determine the effects of different drinking water types on the transcriptional profiles in kidney tissues. Hierarchical clustering analysis showed that the transcriptome profiles from the SPR, PUR, TAP, and DW groups were intermingled, while the gene expression profiles in the DEH group were significantly different from those in all other samples (Fig. 6A). Furthermore, PCA confirmed that the DW, TAP, PUR, and SPR groups showed similar gene expression profiles, and only the DEH group could be readily distinguished from the other groups (Fig. 6B). Taken together, these results suggest that different drinking water types do not induce significantly different biological responses.

## Discussion

The human body can last weeks without food but only days without water. Given that it cannot store water, fresh supplies are needed daily to compensate for losses through the skin, lungs, urine, and feces. Although sufficient water intake is vital to maintaining health, most adults do not hydrate according to the recommended level. Moreover, in adults, the consumption of diuretic beverages, such as caffeinated coffee, tea, and alcohol, is on the rise, resulting in an increased risk of chronic dehydration^2^. Nevertheless, only a few studies have evaluated the effects of long-term inadequate water intake. Although the role of water in the body has been demonstrated based on one or two days of acute complete water deprivation in animal models^37,38^, complete water deprivation is rarely encountered in daily life. Therefore, models of long-term water deprivation may be more applicable. In this study, mice were granted access to water for 15 min/day for a long experimental period of 4 weeks; these conditions have been reported to not cause stress in rodents, as measured by behaviors and serum corticosterone levels^39^. Thus, we observed that the application of this protocol resulted in consumption of approximately half of the water consumed on average by mice in the CON group for a 4-week experimental period; this resulted in a decrease in the body weight of RES mice to 86.7% that of CON mice. In general, 80–85% of initial ad libitum-fed body weight is considered to be the tolerated threshold^40^. In addition to moderate body weight reduction, we demonstrated that the RES regimen caused a significant reduction in urine volume and a dramatic increase in the transcription levels of vasopressin in the brain. Hence, the RES protocol employed in this study sufficiently induced physiological responses to dehydrated conditions. To the best of our knowledge, this is the first study to report the effects of long-term prolonged dehydration on physiological, biochemical, and genomic changes in an in vivo system (Supplementary Fig. S1).

One notable change in our dehydrated mice was a significant increase in serum BUN and creatinine levels compared with CON mice; it has been reported that these two markers are early signs of poor kidneys function^41^. Subsequent analyses therefore focused on changes in kidney tissues at the molecular level. Indeed, the kidney gene expression profiles of RES mice were significantly different from those of CON mice. Genes associated with aquaporins (AQPs), such as *Aqp2*, *Aqp3*, and *Aqp4*, were significantly upregulated in the kidneys of RES mice. AQPs are a family of membrane proteins that function as water-permeable channels. Eight AQPs, including AQP1, -2, -3, -4, -6, -7, -8, and -11, are expressed in different renal segments and various cells, functioning to maintain normal urine concentrations, tissue development, and substance metabolism^42^. Compared with other AQPs, AQP2, -3, and -4 are exclusively expressed in the principal cells of the collecting ducts, which are important tubular segments for regulating body water homeostasis and urine concentrations^35^. Therefore, it is unsurprising that the dysfunction and dysregulation of these AQPs results in various water balance-associated disorders. Numerous studies have demonstrated that the dysregulation of the AQPs that are expressed in renal collecting ducts leads to severe disturbances in water, electrolytes, and acid–base balance, as well as blood pressure disturbances^43^. Particularly, the dysregulation of AQP2-mediated water reabsorption in the collecting ducts is a primary pathophysiological mechanism underlying disease conditions involving body water disturbances, such as systemic water retention or water-losing states^44^. Based on our results, prolonged dehydration due to inadequate water intake led to the dysregulation of *Aqp* gene expression in the kidneys. This suggests that it contributes to illnesses related to disorders of water homeostasis in the body.

Importantly, kidney damage was the most significant biological process category that was over-represented among the differentially expressed genes in the kidneys of RES mice. Among the damage-related mechanisms in the kidneys, the complement system is an innate immune surveillance system that plays key roles in elimination of invading microorganisms as a first line of defense to regulate inflammation^45^. The importance of this system in protection against pathogens is demonstrated by the susceptibility of patients with congenital complement deficiencies to opportunistic infections^46^. Further, patients deficient in complement regulatory proteins, such as factor H (CFH) and factor B (CFB), are predisposed to bacterial and viral infections. Our data showed that the expression of *Cd55*, *Cfh*, and *Cfb* in the complement system was markedly downregulated in RES mice, suggesting the possibility of a significantly higher risk of renal infection following prolonged dehydration. Our data also demonstrated that the natural antioxidant defense system of the kidneys was dysregulated during prolonged dehydration. Indeed, the *Gss*, *Gstp1*, and *Cat* genes, encoding major enzymes involved in defense mechanisms against oxidative stress^47^, were significantly downregulated in RES mice. In fact, patients with CKD typically suffer from chronic inflammation and often have severely impaired antioxidant systems, which progressively worsen with the degree of renal failure^48^. Based on the results obtained in this study, we assumed that persistent dehydration could induce kidney damage owing to the dysregulation of key renal defense systems, such as the anti-inflammatory and antioxidant systems.

Here, we showed that chronic activation of vasopressin and suppression of the NRF2-signaling pathway may play an important role in the deleterious mechanism implicated in kidney damage that is induced by prolonged dehydration. Prolonged dehydration can increase plasma osmolality and trigger activation of the hypothalamic–pituitary–adrenal axis, resulting in a consequent increase in vasopressin secretion^49,50^. Chronically high levels of vasopressin induce morphological and functional changes in the kidney to concentrate urine volume^51^. Sustained secretion of vasopressin can indirectly reduce the efficiency of sodium and urea excretion, while increasing the GFR rate, imposing an excessively increased energetic demand on the kidneys^52^. Moreover, chronic activation of vasopressin can increase levels of ROS, which are detrimental to renal cells and cause oxidative stress, in the kidneys^53^. Thus, in the long-term, such vasopressin-induced changes might continue to burden the kidneys, thereby contributing to the progression of kidney damage. Sustained dehydration also contributed significantly to kidney damage by inducing the suppression of the NRF2-regulated protective defense system against oxidative stress. According to our results, the expression of the antioxidant system transcriptionally regulated by NRF2 was significantly decreased in RES mice. Specifically, the expression of the thioredoxin (TXN) antioxidant system (including TXN, thioredoxin reductase 1 [*Txnrd1*], sulfiredoxin [*Srxn1*]), which is essential for the reduction of oxidized protein thiols, and the glutamate-cysteine ligase (*Gcl*) complex (including *Gclc* and *Gclm*), which regulates intracellular glutathione (GSH) levels, was significantly suppressed in RES mice. In addition, transcription levels of a number of ROS-detoxifying enzymes, such as glutathione peroxidase and several glutathione S-transferases, which use GSH to inactivate ROS and thus lower oxidative stress, were significantly decreased by prolonged dehydration. Mechanistically, long-term dehydration due to insufficient water intake induces chronically high levels of vasopressin secretion to increase renal oxidative stress, as well as suppresses the antioxidant defense system mediated by the NRF2-signaling pathway, ultiamtely leading to kidney damage.

Recently, there has been a considerable increase in public interest regarding drinking water sources. A survey of the literature on consumer preferences suggests that bottled water use is driven by health beliefs and perceptions regarding drinking water^54^. That is, most believe that, compared with TAP, bottled water has additional health benefits and is considered a “healthy option”, even if the consumer is unsure as to why. However, according to our findings, the consumption of bottled SPR and TAP did not result in any differences in physiological responses. These results may have important implications in terms of environmental sustainability. On average, it takes approximately 3 L of regular water to produce 1 L of bottled water, amounting to a wastage of over 100 billion L of water per year^55^. Furthermore, it takes over 2.5 million tons of CO_2_ to produce the amount of bottled water required for US consumption owing to the energy needed for packaging, transportation, and refrigeration^23^. Thus, the production and consumption of bottled water can seriously damage the natural environment and exacerbate climate change^56^. In a comparison based on ecological footprint and carbon footprint, drinking TAP produced footprints almost 285-fold smaller than that associated with the consumption of bottled water^57^. This means that per functional 1.5 L unit, consuming TAP prevents the emission of 259 CO_2_ equivalent grams of greenhouse gases. Therefore, our results can be applied to facilitate sustainability campaigns aimed at discouraging bottled water consumption and promoting TAP consumption. Furthermore, if TAP cannot be consumed, a reusable water filter could be proposed as an eco-friendly alternative to bottled water counterparts. Although major efforts are required, consuming TAP or filtered water rather than bottled water would be substantially beneficial to the aim of becoming carbon neutral so as to ensure sustainability.

Certain limitations were noted in this study. First, while this study has the advantage of demonstrating physiological, biochemical and transcriptional response to prolonged dehydration it was limited by the challenges associated with determining the optimal water intake for healthy individuals. Based on the our data, water intake of 1/2 to 1/3 of normal water intake can significantly increase BUN and creatinine levels among blood biochemical parameters, and can adversely affect the kidneys. Through these results, it can explain the critical minimum water intake amount, however, there is a limiation that it cannot explain the recommended adequate water intake amount. Therefore, further studies are needed to determine in vivo responses to rehydration using rehydration models in mice or even humans. Second, drinking water potentially be contained very low levels of naturally occuring compounds. These compounds present in drinking water, including trihalomethanes (TMHs) which is a disinfection by-product formed through chlorine compounds, could have lasting effects on health. Although this study is a chronic model with a longer period of intervention in drinking water compared to previous studies, there is a limitation in that it cannot be explained whether these compounds will have an effect when different types of water are supplied for a long period of more than 4 weeks. Therefore, further observation of long-term effect of different drinking water types is necessary.

## Conclusion

To the best of our knowledge, this is the first study to investigate the effects of prolonged dehydration in daily life on physiological deterioration, biochemical imbalances, and altered transcriptional networks in the kidneys. Notably, the consumption of drinking water from different sources had little effect on type-specific biochemical responses, ranging from physiological values to molecular levels of transcriptome profiles, in the kidneys of mice. These findings provide novel insights to support the intake of sufficient water to support physiological processes. They also suggest that sufficient water consumption is more critical than consuming a particular type of drinking water. Collectively, these findings serve as a basis for practical action to reduce the consumption of bottled water, thus promoting environmental sustainability.

## Experimental

### Animals and experimental design

This study consisted of two animal studies (*Experiment 1* and *Experiment 2*).

*Experiment 1* After 3 days of acclimation, 7-week-old male C57BL/6J mice (Jackson Laboratory, Bar Harbor, ME, USA) were randomly assigned to the control (CON, n = 20) or water restriction (RES, n = 20) groups. A total of four groups were used in the comparison: CON 2W (n = 10), CON 4W (n = 10), RES 2W (n = 10, early restriction), and RES 4W (n = 10, restriction). The mice were housed at a constant temperature (22 ± 2 °C) with a 12 h/12 h light/dark cycle (lights on from 08:00 am) and were provided *ad libitum* access to an AIN-93G purified diet (Dyets Inc., Bethlehem, PA, USA). Animals in the RES group were maintained on the dehydration schedule until sacrifice.

*Experiment 2* Mice were randomly assigned to five groups, i.e., DW (n = 7), SPR (n = 7), PUR (n = 7), TAP (n = 7), and DEH (n = 7) groups. Specifically, distilled water was used for the DW and DEH groups, bottled Evian spring water (Evian, Evian-les-Bains, France) was used for the SPR group, filtered water from a Coway filtration appliance (Coway Co., Seoul, Korea) was used for the PUR group, and tap water (Seoul, Korea) was used for the TAP group for 4 weeks. Mice in the DEH group were provided access to water for 15 min only per day as described for the RES group in *Experiment 1* for 4 weeks. The other groups were provided water *ad libitum*. All the mice were also fed an AIN-93G purified diet *ad libitum* throughout the study period.

Body weight, food intake, and water consumption were recorded daily, both before and after providing access to water. Further, the daily energy intake was calculated by multiplying the dietary intake (g/day) by the energy density (kcal/g) of the provided diet. To collect urine and feces, some mice were moved into metabolic cages for 5 days before termination of the experiment. After 2 days of acclimation, urine and feces samples were collected from the experimental mice at 24 h intervals for 3 days. On the final day, the mice were fasted for over 12 h and euthanized via an intraperitoneal injection of 20% urethane (U2500; Sigma-Aldrich, St. Louis, MO, USA); blood and tissues were then collected. All experimental procedures were approved by the Institutional Animal Care and Use Committee (IACUC) of Seoul National University (approval no. SNU-130102–3) and were performed in strict accordance with IACUC guidelines for the care and use of laboratory animals. The authors complied with the ARRIVE guidelines.

### Water restriction

Chronic water restriction was induced for 4 weeks as previously described^58^. Briefly, animals in the RES group were given limited access to water; a water bottle was given to animals for 15 min at the same time each day during the experimental period. Conversely, CON mice were provided distilled water ad libitum.

### Blood biochemical analysis

Blood samples collected from the carotid artery of the mice were transferred into tubes without anticoagulant. Thereafter, an aliquot of the blood samples was placed in micro-capillary tubes for hematocrit analysis. Specifically, hematocrit level was determined as the ratio of blood cells per whole blood volume after centrifugation. Further, serum was obtained via centrifugation at 3000 rpm and 4 °C for 20 min to determine the biochemical indices of nutritional status, glucose, total protein, total cholesterol, total bilirubin, blood urea nitrogen, creatinine, glutamate oxaloacetate transferase (sGOT), and glutamate pyruvate transferase (sGPT) levels in serum were determined using a dry-chemistry blood analyzer (Spotchem SP-4410; Arklay, Kyoto, Japan) in accordance with the manufacturer’s instructions.

### Total RNA isolation

Total RNA was extracted from the kidneys of each animal using a DNA-free RNA isolation kit (RNAqueous-4PCR kit; Ambion, Austin, TX, USA) according to the manufacturer’s instructions. Thereafter, total RNA integrity and quantity were assessed using an Agilent 2100 bioanalyzer (Agilent Technologies, Palo Alto, CA, USA).

### Microarray hybridization

For microarray hybridization, only total RNA with optical density 260/280 ratio > 2.0 were used. RNA samples were first amplified for array analyses using an Illumina Total Prep RNA Amplification Kit (Ambion) according to the manufacturer’s instructions. Briefly, 500 ng of total RNA, isolated from kidney tissues, was used to prepare labeled cRNA via overnight incubation according to the manufacturer’s protocol. The quality and quantity of the labeled cRNA were monitored using a Nanodrop 2000 Spectrophotometer. Thereafter, the amplified cRNA (1.5 μg) was hybridized on MouseWG-6 Expression BeadChip arrays, containing over 45,281 well-annotated Ref transcripts, according to the manufacturer’s standard protocol. The beadchips were dried and subsequently scanned on a BeadArray Reader (BeadStation 500G Instrument; Illumina Inc., San Diego, CA, USA). Finally, spot image identification and quantification were performed using Genome Studio Software v1.0.2. (Illumina Inc.).

### Bioinformatic analysis of microarray data

A mouse whole-genome microarray, MouseWG-6 v.2 Expression BeadChips (accession no. GLP6887; Illumina Inc.), was used according to the manufacturer’s instructions. The analysis was performed as previously described^59^. Specifically, the raw data were preprocessed through three steps. Step 1: background correction was performed; Step 2: data were log-transformed to a log2 scale; Step 3: data was normalized using the quantile normalization method corresponding to Genome Studio Software (Illumina Inc.). Differentially expressed genes among the four groups were tested using analysis of variance (ANOVA) at p < 0.01 using Partek Genomics Suite Software v6.6 (Partek, St. Louis, MO, USA). Furthermore, the average gene expression levels were compared between groups (4 W RES vs. 4 W CON), and the calculated p values were corrected for multiple comparisons using an FDR algorithm. Significant genes with fold changes greater than 2 and FDRs less than 0.05 were used for subsequent analyses. Principle component analysis (PCA) and hierarchical clustering analysis were then performed using Genesis Software v1.7.5 based on the Pearson correlation distance matrix with average linkage algorithm. Furthermore, significant functional categories were determined via right-tailed Fisher’s exact tests. To examine the significance of functional categories that were classified based on the Ingenuity Knowledge Base, Gene Set Enrichment Analysis was carried out (http://www.broadinstitute.org/gsea/index.jsp). Mechanistic networks underlying signaling pathways were built based on the Ingenuity Knowledge Base^60^. The functional network map of gene sets was constructed using CytoScape software v3.2.0 (http://cytoscape.org).

### Quantitative RT-PCR

Brain tissue (hypothalamus) were homogenized (IKA, Werge GmbH & Co. KG, Staufen, Germany), and total RNA was extracted using TRIzol reagent (Ambion). This was followed by the DNase I treatment of the total RNA and its conversion to cDNA via a two-step procedure using the MessageSensor RT Kit (Ambion); mRNA levels were quantified using the SYBR-GREEN qPCR method (Applied Biosystems, Waltham, MA, USA). Glyceraldehyde-3-phosphate dehydrogenase (*Gapdh*) was used as a housekeeping gene for the normalization of the mRNA expression corresponding to each sample. The RT-PCR primer sequences for Avp were 5′-CCAGGATGCTCAACACTACG-3′ (forward) and 5′-CTCTTGGGCAGTTCTGGAAG-3′ (reverse), and for *Gapdh*, they were 5′-TGCACCACCAACTGCTTAG-3′ (forward) and 5′-GATGCAGGGATGATGTTC-3′ (reverse). Relative mRNA expression levels (i.e., relative to that of *Gapdh*) were calculated using the ΔΔCT method.

### Statistical analysis

All data were expressed as means ± standard errors of the means. Statistically significant differences between two groups (p < 0.05) were evaluated using unpaired Student’s t-tests, and significant differences among various water source groups were tested by performing one-way ANOVA followed by Duncan’s multiple range test (p < 0.05). All the statistical analyses were performed using Prism 9 software (GraphPad Software, San Diego, CA, USA) and Partek Genomics Suite Software v6.6 (Partek Inc.).

## Supplementary Information

Supplementary Information.

## Data Availability

The data that support the findings of this study are available from the corresponding author upon reasonable request.
