# Supplementary material for: Renal transcriptome profiles in mice reveal the need for sufficient water intake irrespective of the drinking water type
Source: Sci Rep. 2022 Jun 28;12:10911. doi: 10.1038/s41598-022-14815-5 (PMC9240086; doi:10.1038/s41598-022-14815-5)
Supplement: Supplementary file 1 — Supplementary Information. [file 41598_2022_14815_MOESM1_ESM.docx]

**Supplementary information**

Renal transcriptome profiles in mice reveal the need for sufficient water intake irrespective of the drinking water type

Woo-Jeong Shon^1,2^, Mi-Na Park^1^, Jooyoung Lee^1^, Ji-Hee Shin^1^, Dong-Mi Shin^1,2*^

^1^ Department of Food and Nutrition, College of Human Ecology, Seoul National University, Seoul 08826, Republic of Korea

^2^ Research Institute of Human Ecology, Seoul National University, Seoul 08826, Republic of Korea

Correspondence to: [shindm@snu.ac.kr](mailto:shindm@snu.ac.kr)

**This supplementary information file includes:**

**Supplementary Table S1**

**Supplementary Figure S1**

**Supplementary Table S1.** Mineral contents and pH of different water types

| **Mineral content (mg/L)** | **TAP** | **PUR** | **SPR** |
| --- | --- | --- | --- |
| Calcium (Ca) | 24.83 | 0.52 | 76.35 |
| Sodium (Na) | 12.14 | 1.21 | 7.42 |
| Magnesium (Mg) | 4.90 | 0.08 | 27.64 |
| Potassium (K) | 3.08 | 0.18 | 1.53 |
| Silica (SiO2) | 4.73 | 0.30 | 16.59 |
| Copper (Cu) | 0.027 | ND | ND |
| Zinc (Zn) | 0.005 | ND | ND |
| Silicon (Si) | 2.19 | 0.14 | 7.68 |
| Barium (Ba) | 0.02 | ND | 0.117 |
| Boron (B) | 0.01 | ND | 0.01 |
| Strontium (Sr) | 0.117 | ND | 0.401 |
| P, N, Fe, Mn, Ni, Se, Al,  Cr, Ag, PB, Cd, As | ND | ND | ND |
| pH | 7.5 | 6.8 | 7.7 |

Abbreviations: ND, Not detected; PUR**,** purified water; SPR, spring water; TAP**,** tap water.

**
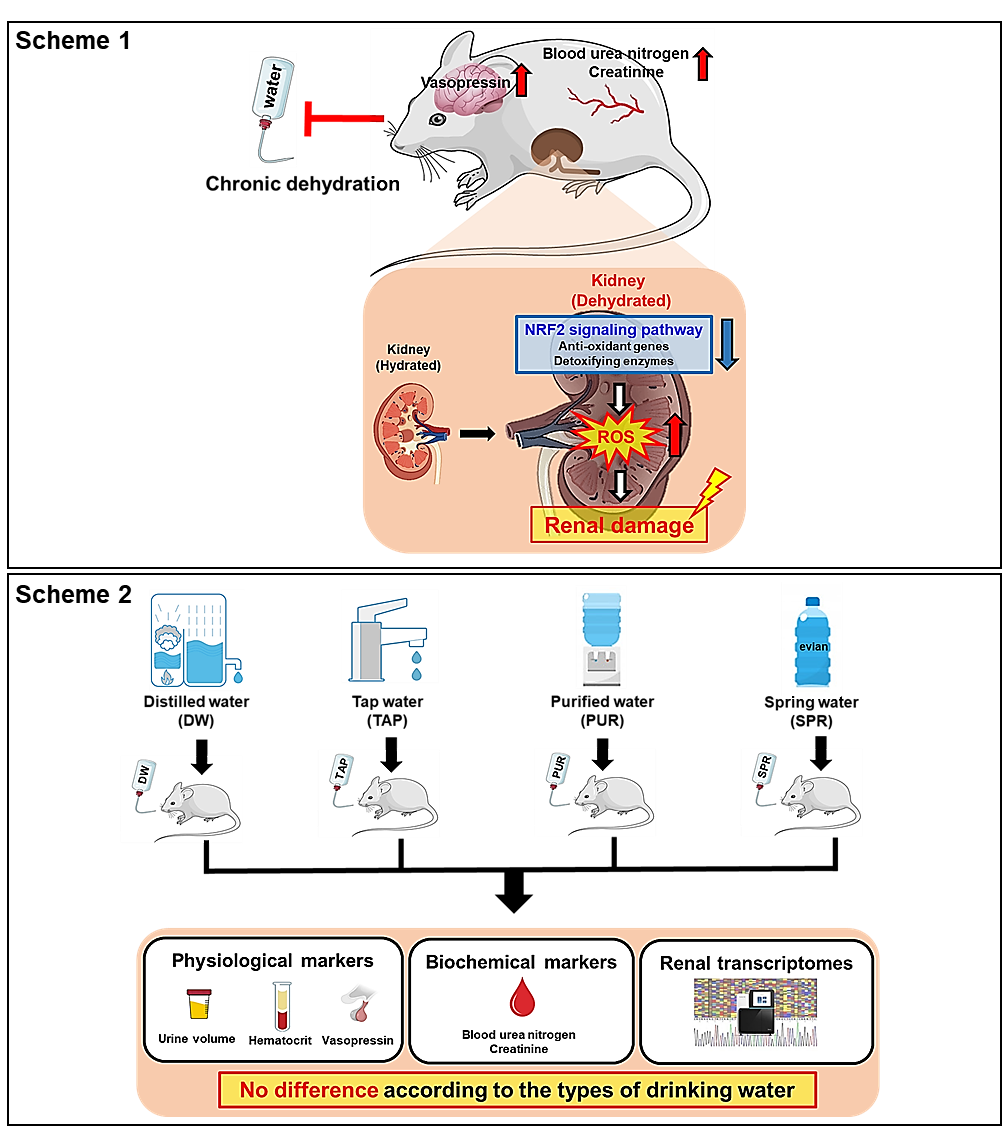
Figure S1.** Schematic depicting that prolonged dehydration induces renal damage by suppressing the NRF2-signaling pathway, thus targeting the cytoprotective system. Moreover, different water sources do not affect specific biological responses. Hence, sufficient water intake is more critical than consuming a specific water type.
